# Oxytocinergic Cells of the Hypothalamic Paraventricular Nucleus Are Involved in Food Entrainment

**DOI:** 10.3389/fnins.2020.00049

**Published:** 2020-01-31

**Authors:** Mario Caba, César Huerta, Enrique Meza, Manuel Hernández, María J. Rovirosa-Hernández

**Affiliations:** ^1^Centro de Investigaciones Biomédicas, Universidad Veracruzana, Xalapa, Mexico; ^2^Doctorado en Ciencias Biomédicas, Centro de Investigaciones Biomédicas, Universidad Veracruzana, Xalapa, Mexico; ^3^Instituto de Neuroetologia, Universidad Veracruzana, Xalapa, Mexico

**Keywords:** food entrainable oscillator, supraoptic nucleus, food-intake, sympathetic system, parasympathetic system, food anticipatory behavior, pancreas

## Abstract

When food is presented at a specific time of day subjects develop intense locomotor behavior before food presentation, termed food anticipatory activity (FAA). Metabolic and hormonal parameters, as well as neural structures also shift their rhythm according to mealtime. Food-entrained activity rhythms are thought to be driven by a distributed system of central and peripheral oscillators sensitive to food cues, but it is not well understood how they are organized for the expression of FAA. The hormone Oxytocin plays an important role in food intake, satiety and homeostatic glucose metabolism and although it is recognized that food is the main cue for food entrainment this hormone has not been implicated in FAA. Here we investigated the activity of oxytocinergic (OTergic) cells of the hypothalamus in relation to the timing of feeding in rabbit pups, a natural model of food entrainment. We found that OTergic cells of the supraoptic nucleus and the main body of the paraventricular nucleus (PVN) are activated after feeding which suggests that OT may be an entraining signal for food synchronization. Moreover, a detailed analysis of the PVN revealed that OTergic cells of the caudal PVN and a subpopulation in the dorsal part of the main body of this nucleus shows activation *before* the time of food but not 12 h later. Moreover this pattern persists in fasted subjects at the time of the previous scheduled time of nursing. The fact that those OTergic cells of the dorsal and caudal part of the PVN contain preautonomic cells that project to the adrenal, pancreas and liver perhaps may be related to the physiological changes in preparation for food ingestion, and synchronization of peripheral oscillators, which remains to be determined; perhaps they play a main role in the central oscillatory mechanism of FAA as their activity persists in fasted subjects at the time of the next feeding time.

## Introduction

In the brain several structures show rhythms coupled to the light/dark cycle including the paraventricular hypothalamic nucleus (PVN). Retrograde tracing studies indicate that the PVN is a target output from the suprachiasmatic nucleus (SCN) and they show a parallel phase-dependent induction of Fos protein, the product of the *c-Fos* gene, in response to a light pulse (Munch et al., 2002) and explants of this nucleus in culture do not show circadian rhythmicity (Abe et al., 2002). Overall the above results indicate that the activity of the PVN is rhythmic but is driven by the SCN. In the intact rat cells showing Fos expression in the PVN increase in numbers during the night in comparison to the day (Nunez et al., 1999). It is widely recognized that this time keeping mechanism generated from the SCN to other brain structures as the PVN, is present in several central and peripheral organs to ensure that bodily processes are carried out at the appropriate, optimal time of day or night (Kriegsfeld and Silver, 2006). However, when subjects are exposed to food for a few hours daily, this orderly hierarchy uncouples from the SCN. Animals develop an increase in locomotor behavior a few hours before food presentation, which is termed food anticipatory activity (FAA; Mistlberger, 1994). Other metabolic and hormonal parameters as well as central structures are also entrained by timing of food, even in the absence of the SCN (Caba and Mendoza, 2018). Food-intake then is a cue that elicits physiological responses, which act as entrainment stimuli for the brain and peripheral organs (Escobar et al., 2009; Mistlberger, 2011). However, despite considerable research effort, the internal entrainment stimuli are not well understood nor how central and peripheral oscillators are coupled (Mistlberger, 2011). In the present contribution we explored the oxytocinergic (OTergic) system of the hypothalamic supraoptic (SON) and PVN, the main sources of OT in the brain (Swanson and Kuypers, 1980) in relation to food-entrainment.

In contrast to the low activity of the PVN during the day (Nunez et al., 1999) this nucleus shows a sharp increase in Fos protein, after food presentation during the day in food entrained adult rats (Angeles-Castellanos et al., 2004). This result is interesting because it is possible that this increased activity of the PVN could be part of an afferent system that brings information about food intake; however, their phenotypical identity has not yet been explored. In present contribution we explored OTergic cells of both SON and the PVN 12 h before, and immediately before and after food ingestion by using the rabbit pup, which is considered a natural model of food restriction (Caba and Mendoza, 2018). Whether nursing occurs during the day or the night, pups show intense FAA before arrival of the mother (Caba et al., 2008). In the rabbit pup, metabolic, and physiological parameters such as corticosterone, and neural activity in some structures, shift their rhythm in relation to nursing time, similar to rodents where mealtime can act as a zeitgeber (Morgado et al., 2008, 2010; Caba and Mendoza, 2018). We found a differential activation of OTergic cells in different subregions of the PVN in relation to timing of feeding. This perhaps could be related to a possible main role of OT cells of the PVN both as an entraining signal and as an important pathway for the coordination between central and peripheral structures for the organized oscillation of the system that leads to the food entrainment phenomenon, which remains to be determined.

## Materials and Methods

### Animals and Housing

New Zealand White female rabbits bred in our colony in Xalapa, Mexico, were maintained under controlled light (12 h light; 12 h dark) and temperature (24 ± 1°C) conditions. Rabbit pellets (Purina) and water were provided *ad libitum*. Rabbits were exposed to sexually experienced males, mated and housed individually in stainless steel cages and were monitored daily from day 28 of pregnancy until delivery. Cages had two compartments, one for the mother and one for the pups (width, 0.60 m; length, 0.50 m; height, 0.40 m; each cage); the two compartments are linked by a tunnel with slide doors at each end, that permit the experimenter to control the access of the mother to the nest (Caba et al., 2008). On the last days of pregnancy the rabbits were provided with straw to build the nest where pups were born and litters were adjusted to four to five pups. To determine that pups were entrained by timing of nursing locomotor activity was monitored by an infrared sensor located on the ceiling of the nest (Caba et al., 2008; Morgado et al., 2010). Experiments and animal handling were conducted according to the national guide for care and use of animal experimentation approved by the Ethics Committee of Universidad Veracruzana, in accordance with the procedures of the National Guide for the Production, Care and Use of Laboratory Animals (Norma Oficial Mexicana NOM-062-ZOO-1999), which complies with international guidelines laid down by the Society for Neuroscience.

### Experimental Design

On the day of parturition (PD0), the door between the mother’s compartment and the tunnel was closed and starting the next day (PD1) was opened for nursing every day at 10:00 am until PD7. The doe entered the nest immediately and the mean duration of nursing was 230 ± 7 s (Mean ± SE). On PD7 pups were sacrificed just before nursing (N10:00), at 1.5 h (N11:30) or 12 h (N22:00) following nursing (*n* = 4 at each time point). To explore persistence of possible oscillations additional pups were fasted for two nursing bouts, as in previous experiments (Morgado et al., 2008; Moreno et al., 2013) and sacrificed on PD9 at their previous scheduled time of nursing (F10:00) or 12 h later (F22:00).

### Immunohistochemistry

Subjects were euthanized with an overdose of sodium pentobarbital (20 mg per pup, intraperitoneal) and were perfused transcardially with saline solution (0.9%) followed by 4% paraformaldehyde in phosphate buffer (PB; 0.1 M, pH 7.4). The brains were collected, cryoprotected (Caba et al., 2008) and sectioned coronally at 50 μm with a cryostat (Microm, Walldorf, Germany). Serial sections were collected in PB and one of four sections were labeled sequentially with a primary antibody against Fos protein (1:5,000; sc-52G, Santa Cruz Biotechnology, Santa Cruz, CA) and then against oxytocin (1:5,000; MAB5296, MERCK) following protocols previously established in our laboratory for double label immunohistochemistry of Fos and OT in the rabbit brain (Caba et al., 2003, 2008). In all cases tissue sections of subjects from the different conditions were processed together.

### Cell Counting

**FIGURE 1:**
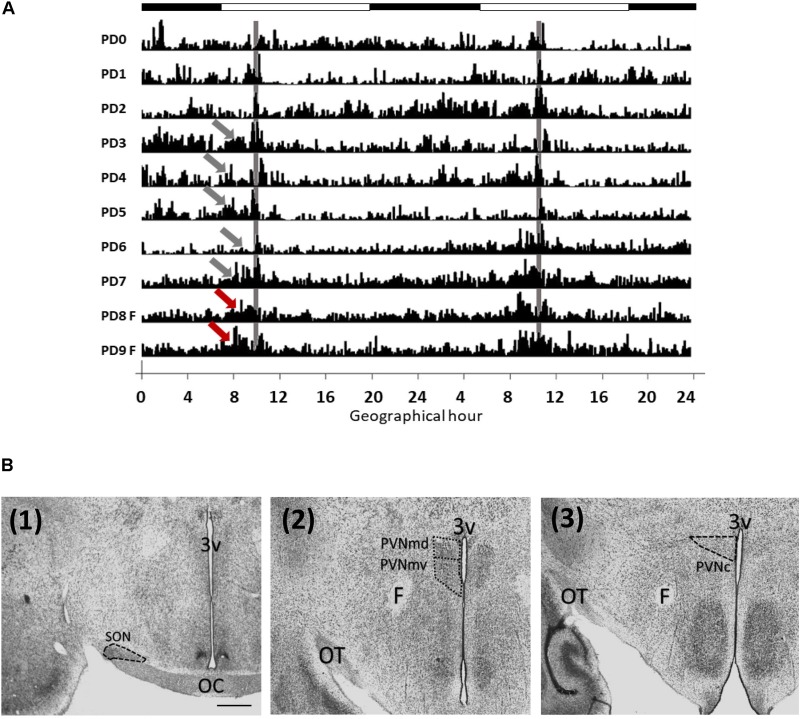
Locomotor activity of rabbits from postnatal days 1 to 9 **(A)** and analyzed areas **(B)**. **(A)** Double plotted actogram from a representative litter. Intensity of activity is represented by black vertical lines. Pups increase their activity (gray arrows) before nursing time (gray vertical line), at PD8 and PD9 the doe was not permitted to nurse but locomotor behavior increased again at the previous scheduled time of nursing (red arrows). Each horizontal line represents a postnatal day. Black and white bar at top represents light/dark cycle of the mother. **(B)** Photomicrographs (Thionin stain) showing the location of the SON (1), PVNmd and PVNmv (2) and PVNc (3). 3v, third ventricle, OC, optic chiasma; OT, optic tract; F, fornix; Scale bar: 500 μm.

In order to quantify Fos- and OT-immunoreactivity (Fos-ir; OT-ir), sections were matched at the same level in all groups. The SON was explored at level NA1 and two levels of the PVN were explored, the main body (PVNm) and their posterior caudal (PVNc) part at levels NP 0.5 and NP 1.5, respectively, of a rabbit brain atlas (Girgis and Shih-Chang, 1981). As we detected a differential activation of the dorsal and ventral part of the PVNm at the different time points explored then those subregions were identified, respectively, as PVNmd and PVNmv. Number of OT- and Fos-/OT-ir cells was quantified in an area of 21621.9, 146964.6, 210961.5, and 39936.5 μm^2^ in the SON, PVNmd, PVNmv, and PVNc respectively (Figure 1B). Two observers blind to the experimental condition of subjects counted cells unilaterally. As shown in the figures single Fos-ir was scarce in all conditions and was not considered in the counting. Images were captured with an image system (Image Pro Plus version 5, Media Cybernetics, Silver Spring, MD, United States) attached to a light microscope (Olympus BX41; Tokyo, Japan).

### Statistics

Locomotor behavior data were recorded and analyzed by an Enright periodogram with the SPAD9 (Omnialva, Mexico) circadian recording system, as in our previous publications (Caba et al., 2008; Morgado et al., 2008). 2-h bins of activity prior to daily nursing were compared with the remaining 22-h bins after nursing by using a Student’s *t*-test for PD 5–7. Analysis of the number of OT-ir and Fos-/OT-ir cells in nursed groups was performed by one-way ANOVA to determine whether there were differences across different time points, followed by a *post hoc* analysis with a Tukey test (R Core Team, 2019). Comparisons between the two different time points on fasting groups were performed using a Student’s *t*-test. Probability levels of *P* < 0.05 were considered significant (R Core Team, 2019). Values are given as means ± SE.

## Results

### Locomotor Activity

Pups developed FAA before nursing as shown in actogram in Figure 1A. Locomotor activity prior to daily nursing was significantly higher than after nursing [*t*(8 = 3.001, *P* = 0.017)]. At PD8 and PD9 pups were fasted but locomotor behavior increased again at the previous scheduled time of nursing. Periodogram indicates a circadian component (*X*^2^, *P* < 0.05).

### OT in PVNm

**FIGURE 2:**
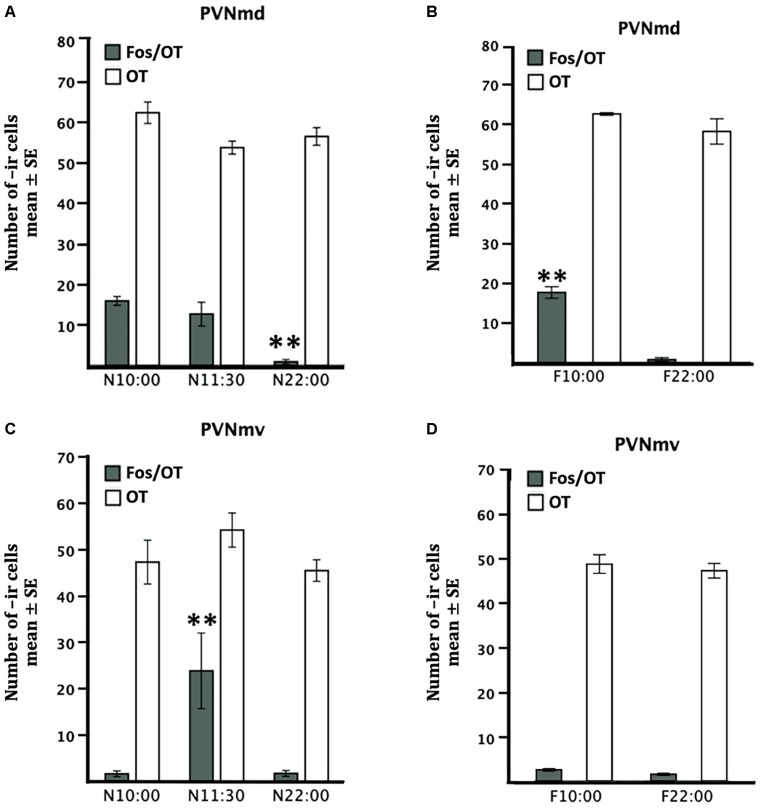
Comparative distribution of OT- and Fos/OT-ir cells in PVNmd **(A,B)** and PVNmv **(C,D)** of pups nursed **(A,C)** and fasted for two nursing bouts **(B,D)**. Nursed pups were perfused just before nursing (10:00) or 1.5 and 12 h later. Fasted pups were perfused at the time of their previous scheduled time of nursing (10:00) or 12 h later. Values are mean ± SE. ^∗∗^*P*<0.01, denotes significant difference between highest and lower Fos-/OT-ir cells between different time points.

Quantitative analysis indicated no significant differences in OT-ir cells in the PVNmd among the three nursed groups [*F*(2,9 = 3.92, *P* > 0.05) Figure 2A] or in the two fasted groups [*t* (3 = 1.46, *P* > 0.05) Figure 2B]. Also there was no significant difference in the PVNmv between nursed [*F*(2,9 = 1.55, *P* > 0.05) Figure 2C] or fasted groups [*t*(3 = 0.63, *P* > 0.05) Figure 2D].

### Fos/OT in PVNm

**FIGURE 3:**
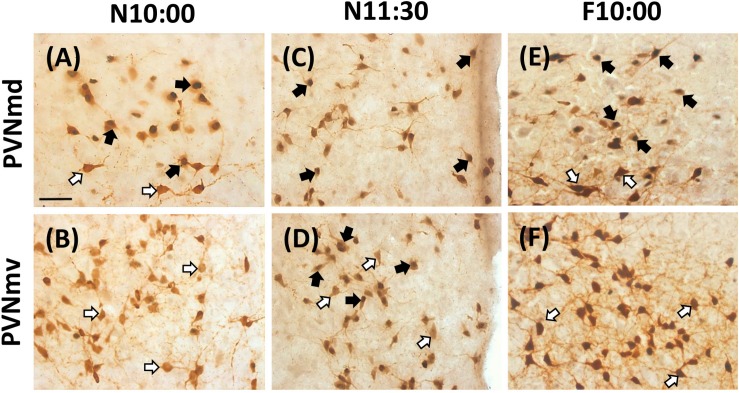
Expression of OT- and Fos-ir in PVNmd and PVNmv in nursed (N10:00 and 11:30) and fasted (F10:00) subjects. Photomicrographs of representative sections of PVNmd **(A)** and PVNmv **(B)** just before nursing (10:00) and 1.5 **(C,D)** h after. Note an increase in double labeled Fos-/OT-ir cells in the NPVmd **(A)** just before nursing, which persist in fasted subjects at the time of the previous scheduled time of nursing **(E)**. On the contrary a similar increase is observed in the PVNmv 1.5 h but after **(D)**, not before **(B)** nursing, which does not persist in fasting **(F)**. Scale bar, 50 μm.

The two subregions of the PVNm, PVNmd and PVNmv showed a different pattern in the number of Fos-/OT-ir cells in the different conditions (Figure 2). The PVNmd showed a significant increase of Fos-/OT-ir double-labeled cells before nursing that persisted in fasted subjects at the time of the previous scheduled time of nursing. In contrast the PVNmv only showed a significant increase of double labeled cells after nursing. Quantitative analysis indicates that Fos/OT-ir cells in the PVNmd varied significantly among before, after and 12 h after suckling [*F*(2,9 = 18.57, *P* < 0.001)]; the highest value was observed in N10:00 group in comparison to N22:00 group (*P* < 0.001; Figure 2A); additionally, there were no differences between N10:00 and N11:30 group (*P* > 0.05). The F10:00 fasted group had significant higher number of Fos-/OT-ir cells [*t*(3 = 11.14, *P* < 0.001)] in comparison to F22:00 group (Figure 2B). Quantitative analysis of ventral subregion (PVNmv) indicates a significant difference between groups [*F*(2,9 = 7.26, *P* < 0.01); there was a significant increase in N11:30 group compared to N10:00 and N22:00 (*P* < 0.01 in both cases; Figure 2C). There was no significant difference between the two fasted groups [*t*(3 = 2.44, *P* > 0.05; Figure 2D)]. In Figure 3 we present photomicrographs of representative sections of PVNmd (Figures 3A,C,E), showing an increase in Fos-/OT-ir cells before nursing (Figure 3A) which persist in fasted subjects (Figure 3E). In contrast PVNmv shows an increase in Fos-/OT-ir cells only after nursing (Figure 3D).

### OT in PVNc

**FIGURE 4:**
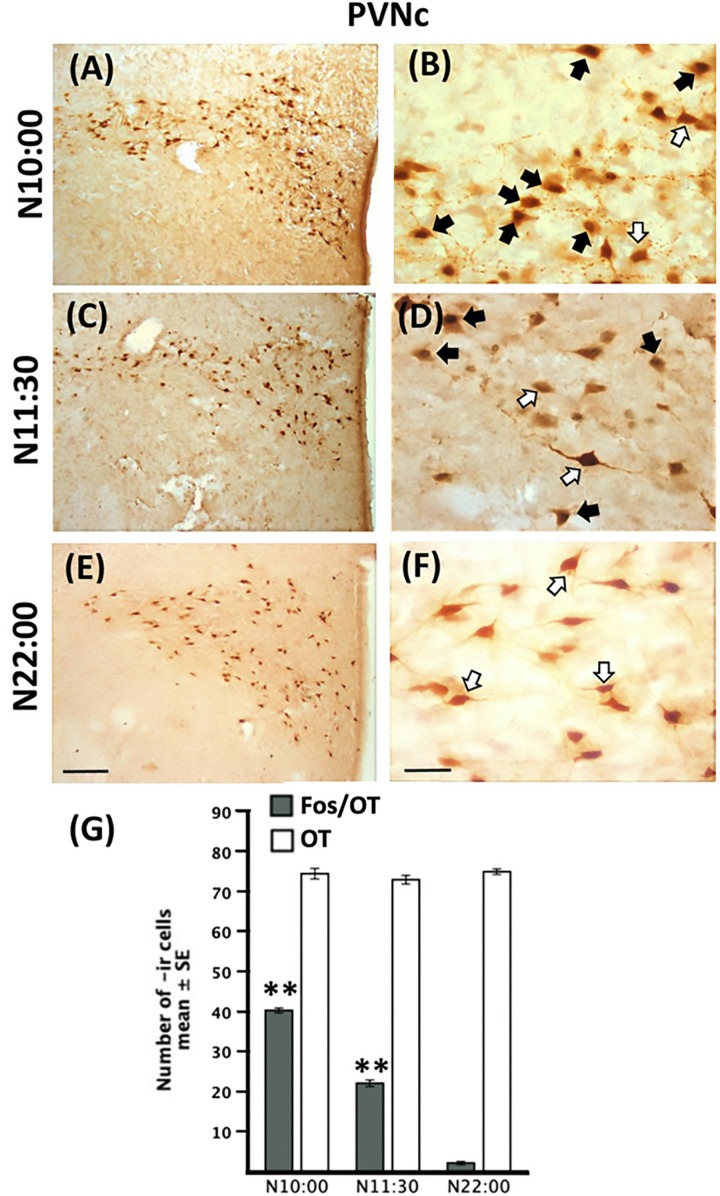
Induction of Fos-/OT-ir cells in PVNc before nursing time. Photomicrographs of representative sections of the PVNc at the time of nursing (10:00, **A**), 1.5 **(C)** and 12 **(E)** h after nursing and magnifications of the respective time points **(B,D,F)**. Note the increase of Fos-/OT-ir cells just before **(A,B)** but not 12 h after **(E,F)** nursing. **(G)** Quantitative analysis of OT- and Fos-/OT-ir at the time of nursing (N10:00) and 1.5 (11:30) and 12 h (N22:00) h after. White arrow, OT-ir; black arrow Fos-/OT-ir. ^∗∗^*P*<0.01, denotes significant difference between Fos-/OT-ir cells between N10:00 and N11:30 groups in comparison to N22:00 group. Scale bar **(A,C,E)**, 100 μm; **(B,D,F)**, 50 μm.

**FIGURE 5:**
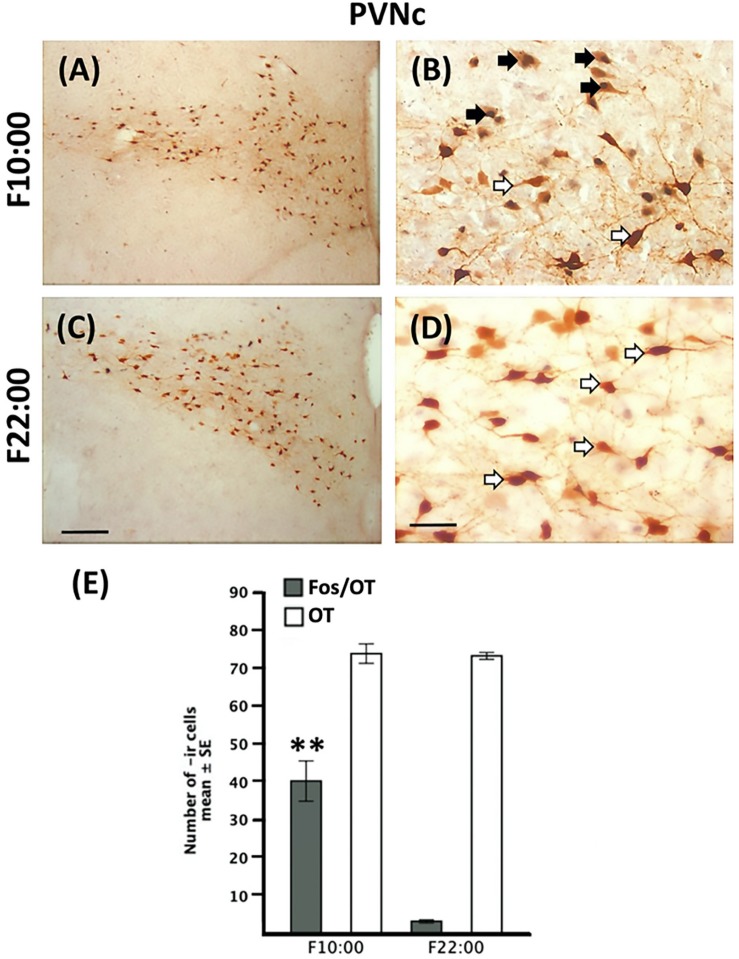
Persistence of Fos-/OT-ir cells in the PVNc in fasted subjects. Photomicrographs of representative sections of the PVNc of fasted subjects at the time of previous scheduled time of nursing **(A)** and 12 h after **(C)** and magnifications of the respective time points **(B,D)**. Note the increase of Fos-/OT-ir cells at the time of previous scheduled nursing **(A,B)** in fasted subjects and a decrease 12 h later **(C,D)**. **(E)** Quantitative analysis of OT- and Fos-/OT-ir cells in fasted subjects at the time of previous scheduled time of nursing (F10:00) and 12 h (F22:00) later. White arrow, OT-ir; black arrow Fos-/OT-ir. ^∗∗^*P* < 0.001, denotes significant difference between F10:00 and F22:00 Fos-/OT-ir cell groups. Scale bar **(A,C)**, 100 μm; **(B,D)**, 50 μm.

Quantitative analysis revealed no significant difference in the number of OT-ir cells in the PVNc in any of the nursed [*F*(2,9 = 0.95, *P* > 0.05) Figure 4G] or fasted [*t*(3 = 1.64, *P* > 0.05) Figure 5E] groups.

### Fos/OT in PVNc

In Figures 4A–F we present photomicrographs of representative sections of PVNc. Quantitative analysis indicated that Fos-/OT-ir in PVNc in nursed subjects varied significantly among before, after and 12 h after suckling [*F*(2,9 = 893.3, *P* < 0.001) Figure 4G]; the highest value in N10:00 group was significantly different in comparison to N11:30 and N22:00 groups (*P* < 0.001 in all cases; Figure 4G). Additionally value at N11:30 was significantly higher than that of N22:00 group (Figure 4G). In relation to fasted subjects the F10:00 group presents significant higher numbers of Fos-/OT-ir cells [*t*(3 = 29.41, *P* < 0.001)] compared to F22:00 group (Figure 5E).

### OT in SON

**FIGURE 6:**
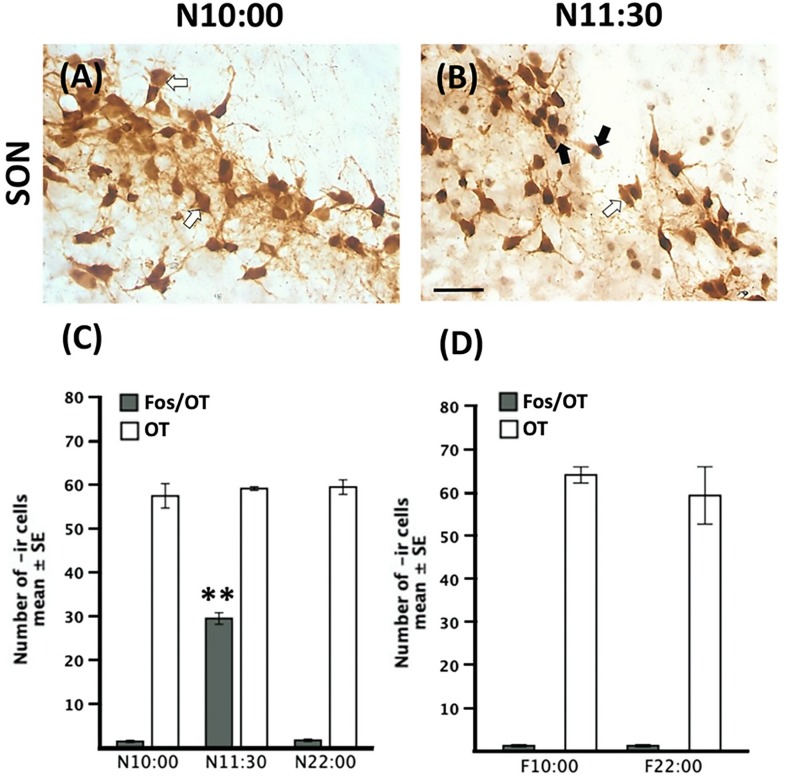
Increase of Fos-/OT-ir cells after nursing in the SON. Representative photomicrographs of the SON just before (N10:00, **A**) and after (N11:30, **B**) nursing. **(C,D)** Quantitative analysis of OT- and Fos-/OT-ir cells in nursed **(C)** and fasted subjects **(D)**. White arrow, OT-ir; black arrow Fos-/OT-ir. Note the increase in double labeled cells only after food ingestion. ^∗∗^*P* < 0.001, denotes significant difference between N11:30 and N:10:00 and N22:00 groups. Scale bar 50 μm.

Quantitative analysis indicated no significant differences between nursed [*F*(2,9 = 0.33, *P* > 0.05)] or fasted [*t*(3 = 0.64, *P* > 0.05) Figure 6] groups.

### Fos/OT in SON

In Figures 6A,B we present photomicrographs of representative sections of SON. Quantitative analysis revealed significant changes in the number of Fos-/OT-ir cells [*F*(2,9 = 494.8)]; there was a significant increase in nursed group (N11:30) compared to the remaining ones (*P* < 0.001 in all cases; Figure 6C). There was no significant difference in the two fasted groups [*t*(3 = 0.00, *P* = 1.00) Figure 6D].

## Discussion

The main findings of the present study are: (1) There is an activation of OT neurons after feeding in the SON and PVNmv, (2) OT cells of the PVNc and in the dorsal part of PVNm shows activation *before* feeding, and moreover, this activation *persists* in fasted subjects at the previous scheduled time of nursing. The PVN is a complex neural structure that integrates essential endocrine and autonomic responses. In the rat it is comprised of several distinct morphological and neurochemical heterogeneous populations of magnocellular and parvocellular cells organized in 10 structural subdivisions (Koutcherov et al., 2000). Magnocellular regions of the SON and some regions of the PVN contain OT or vasopressin (AVP) producing cells that project to the posterior pituitary to release these peptides into the bloodstream (Swanson and Kuypers, 1980). However, parvocellular regions are more complex as they project to other forebrain regions as well as to the brainstem and spinal cord enabling them to influence the activity of the autonomic nervous system (Swanson and Kuypers, 1980). Moreover, their phenotypical identity is more diverse; they contain dopamine, somatostatin, encephalin or corticotrophin-releasing factor, besides OT or AVP (Koutcherov et al., 2000). The cytoarchitectonic subdivisions of the PVN are well characterized in rodents, particularly in the rat (Swanson and Kuypers, 1980), but not in others as in humans (Koutcherov et al., 2000). Also, in the rabbit this nucleus has a loose organization without any obvious subdivision (Schimchowitsch et al., 1989).

As shown in Figure 3C, the double label study indicates that most OT cells of the PVNmv are inactive before feeding but milk ingestion induces Fos protein in many of these cells. On the contrary OT cells from the PVNmd and PVNc show activation *before* the nursing time. This differential activation of OTergic cells in the different experimental conditions suggests a distinct function. By comparison with rat studies, the activation of OT cells in the PVNmv and the SON, is probably more related to an endocrine response to milk intake as food ingestion induces a massive release of OT into the bloodstream (Verbalis et al., 1996) to act peripherally to control metabolic homeostasis (Onaka and Takayanagi, 2019). OT improves insulin sensitivity, increases lipolysis, enhances glucose uptake and lipid utilization in adipose tissue and skeletal muscle and plasma OT levels are notably lower in obese individuals with diabetes (Spetter and Hallschmid, 2017; Ding et al., 2019). However, there is also release of OT in the hypothalamus in regions involved in food intake and energy expenditure as the dorsomedial, arcuate and ventromedial nuclei and the lateral hypothalamus, which contain oxytocin receptors (Onaka and Takayanagi, 2019). In general OT is considered an anorexigenic hormone that inhibits food intake and promotes satiety after food ingestion (Arletti et al., 1989; Olson et al., 1991; Onaka and Takayanagi, 2019).

Notwithstanding the importance of OT in food intake, satiety and homeostatic metabolism this hormone had not been involved in the phenomenon of synchronization by food. The concept of food entrainment includes an afferent pathway that entrains distributed central and peripheral oscillators that drives food anticipatory rhythms (Mistlberger, 2011). In this regard, food ingestion induces a large activation of both Fos protein (Angeles-Castellanos et al., 2004) and *c-Fos* mRNA (Poulin and Timofeeva, 2008) in the main body of the PVN in adult rats under food restriction. In the rabbit model we previously found a similar activation of cells in the main body of the PVN after their only daily period of milk ingestion, which were identified as OTergic cells (Caba et al., 2003), similar to present results. This OT activation was a consequence of food ingestion, as it was not observed in un-nursed pups. In considering that food seems to be the entraining cue for the phenomenon of food entraining in rats (Mistlberger, 2011) and the rabbit pup (Morgado et al., 2011) we propose that those OTergic cells of the main body of the PVN and SON, may play a main role as an entraining cue.

Additionally, our detailed observation of the PVN at different time points of feeding in nursed and un-nursed subjects led us to identify that two subpopulations of OT cells one in the dorsal part of the main body of PVN and those of PVNc, unlike OT cells of the PVNmv, are active before their only period of food ingestion, i.e., they “anticipate” feeding time. Moreover their activation is not permanent, as it is not observed 12 h after feeding although it is observed again at the previous scheduled feeding time even when food was omitted in un-nursed pups. This activation before feeding time agrees with a previous publication in rats. Poulin and Timofeeva (2008) reported an increase of *c-Fos* mRNA in the mediodorsal subdivision of the PVN just before food intake. In the corresponding figure (see Figure 6 in Poulin and Timofeeva, 2008) it is possible to identify that this region lies in the dorsal portion of the PVN, at a similar level identified as PVNmd in our results. We propose that the activation of these PVN cells that anticipate feeding time could be related to the peripheral metabolic changes observed in food entraining (Escobar et al., 2009) through indirect projections to peripheral organs. It is important to consider that PVN as well as SON are the main sources of AVP in the hypothalamus and future experiments are warranted to explore the potential participation of this hormone in food entrainment.

Classical studies demonstrate that PVN OT neurons besides the pituitary also projects to the spinal cord and brainstem regions as the dorsal vagal complex (Swanson and Kuypers, 1980; Sofroniew, 1985). Further studies of denervation of parasympathetic and sympathetic nerves combined with injection of pseudorabies virus (PRV), a transynaptic tracer, in the pancreas, revealed that second order neurons in several brain regions including the hypothalamus and cerebral cortex support a role of these central structures in the endocrine activity and cephalic response of the pancreas to food (Buijs et al., 2001). Furthermore, these authors, by using a similar strategy, also found preautonomic OTergic neurons in the posterior part of the PVN neurons that project to sympathetic or parasympathetic motor nuclei connected with the adrenal and liver, besides the pancreas (Buijs et al., 2003). In agreement, *in vivo* studies demonstrate that activation of sympathetic or parasympathetic innervation of the liver as well as pharmacological manipulation of preautonomic PVN neurons affects glucose homeostasis (O’Hare and Zsombok, 2016). These studies are very interesting in considering that in subjects under food restriction there is a shift in several hormonal and metabolic parameters to timing of feeding. Under this condition before food intake subjects show an increase in corticosterone, free fatty acids and glucagon and a decrease in glycogen and insulin, indicating they are in a catabolic state (Díaz-Muñoz et al., 2000). Moreover, authors also detected an oxidized condition of the liver before food access that suggests a preparatory process to the upcoming food intake (Díaz-Muñoz et al., 2000). In the rabbit pup we previously reported an increase in corticosterone and free fatty acids and a decrease in glycogen, which agree with the proposed catabolic state of subjects before food intake (Morgado et al., 2008, 2010). In considering the anticipatory changes in the liver and pancreas and the control of these organs by the sympathetic and parasympathetic system it is possible that the activated OT cells in PVNmd and PVNc of our present study could play an important role in the physiological preparation during FAA. In addition, in the rat lesions of the catecholaminergic input to the PVN suppress the anticipatory corticosterone peak in food entrained rats (Honma et al., 1992) which may indicate that the observed anticipatory activation of PVN cells could also be a response to a factor released in advance of mealtime. Then we propose that those OT subpopulations can be an important link between the brain and the peripheral organs, as part of the putative multi oscillatory mechanism of the FEO. The facts that in un-nursed pups these cells are active again (present results) together with metabolic and hormonal parameters at the scheduled previous time of nursing (Morgado et al., 2008, 2010), strongly reinforce this assumption. Finally in spite of the possible importance of the PVN cells in food entrainment, lesions of this nucleus indicates that the PVN it is not necessary for food anticipatory activity rhythms (Mistlberger and Rusak, 1988), similar to other brain nuclei (Mistlberger, 2011).

The SON could be more related to the homeostatic actions of OT at peripheral level in considering that this nucleus mainly projects to the posterior pituitary (Swanson and Kuypers, 1980; Sofroniew, 1985). Other hormones as corticosterone, ghrelin, leptin, and glucagon are entrained or seem to play a modulatory role in food entrainment (Patton and Mistlberger, 2013). On basis of present results we consider that is worth to explore OTergic cells in detail by discrete lesion or stimulation (Atasoy et al., 2012) of particular subpopulations of the PVN as they can contribute to the understanding of the food-entraining phenomenon. Additionally, food-entrainment implicates a daily reward mechanism that involves dopamine (Gallardo et al., 2014). On this regard it is interesting that oxytocin regulates feeding also through mechanisms in reward related areas as the nucleus accumbens, amygdala, bed nuclei of the stria terminalis and ventral tegmental area, which contain oxytocin receptors (Lawson et al., 2019).

## Summary and Conclusion

OTergic cells of the SON and PVNmv may play a role as an entraining signal for synchronization by food. Besides discrete populations of OT cells in PVNmd and PVNc can be a link between neural and hormonal and metabolic changes through projections of the autonomic nervous system to prepare subjects during FAA to optimize food ingestion and use of nutrients during the short period of food presentation. Moreover, the persistence of activation of these OT cells at the time of the scheduled next nursing time in un-nursed subjects suggests a self-sustaining oscillation, which perhaps plays a main role in the multioscillatory system in control of food-entrained rhythms.

## Data Availability Statement

The raw data supporting the conclusions of this article will be made available by the authors, without undue reservation, to any qualified researcher.

## Ethics Statement

The animal study was reviewed and approved by the Ethics Committee of Universidad Veracruzana.

## Author Contributions

MC and EM conceived and designed the experiments, and wrote the manuscript. EM, CH, MH, and MR-H performed the experiments. MC, EM, CH, and MH analyzed the data. MR-H, CH, and MH contributed with reagents, design, and elaboration of figures.

## Conflict of Interest

The authors declare that the research was conducted in the absence of any commercial or financial relationships that could be construed as a potential conflict of interest.
